# Impact of Different Carbon Sources on Volatile Organic Compounds (VOCs) Produced during Fermentation by *Levilactobacillus brevis* WLP672 Measured Using Proton Transfer Reaction Time-of-Flight Mass Spectrometry (PTR-ToF-MS)

**DOI:** 10.3390/molecules29143275

**Published:** 2024-07-11

**Authors:** Sarathadevi Rajendran, Iuliia Khomenko, Patrick Silcock, Emanuela Betta, Franco Biasioli, Phil Bremer

**Affiliations:** 1 Department of Food Science, University of Otago, P.O. Box 56, Dunedin 9054, New Zealand; sarathadevi.rajendran@postgrad.otago.ac.nz (S.R.); phil.bremer@otago.ac.nz (P.B.); 2 Sensory Quality Unit, Research and Innovation Centre, Fondazione Edmund Mach, 38098 Trento, Italy; iuliia.khomenko@fmach.it (I.K.); emanuela.betta@fmach.it (E.B.); franco.biasioli@fmach.it (F.B.); 3 Department of Agricultural Chemistry, Faculty of Agriculture, University of Jaffna, Kilinochchi 44000, Sri Lanka; 4 ONFoods-Research and Innovation Network on Food and Nutrition Sustainability, Safety and Security-Working ON Foods, 43121 Parma, Italy

**Keywords:** carbon sources, defined medium, lactic acid bacteria, volatile organic compounds

## Abstract

Bacterial fermentation is considered to be a cost-effective means of generating desired flavour compounds from plant-based substrates. However, the wide range of substrates present in plants makes it challenging to understand how individual components impact on flavour volatile organic compound (VOC) production. To simplify this, a defined medium can be used to better understand VOCs production with regard to individual compounds. In the current study, the VOCs produced by the lactic acid bacterium, *Levilactobacillus brevis* WLP672, growing in a defined medium containing different carbon sources (either glucose (DM), fructose (DMFr) or citrate (DMCi)) under a range of fermentation conditions (time: 0, 7, and 14 days; and temperature: 25 and 35 °C) were assessed using proton transfer reaction time-of-flight mass spectrometry (PTR-ToF-MS). Among the detected mass peaks (*m*/*z*), after 7 days of fermentation, the concentrations of *m*/*z* 45.033 (t.i. acetaldehyde), *m*/*z* 49.011 (t.i. methanethiol), and *m*/*z* 89.060 (t.i. ethyl acetate) were significantly (*p* < 0.05) higher in DM at 35 °C than all other treatments at either temperature. The knowledge obtained will help to produce desirable LAB fermentation flavour VOCs or VOC mixtures that could be used in developing plant-based analogues with acceptable sensory properties.

## 1. Introduction

Consumers, worldwide, are consciously opting to eat more plant-based foods. This shift to a more plant-based diet is driven by a number of factors, including the health benefits of reduced risk of obesity, cardiovascular disease, type II diabetes, hypertension, coronary artery disease, and certain types of cancer, as well as concern for animal welfare, and a wish to reduce the environmental footprint of their diet [1,2]. This desire to eat more plant-based foods has led to an increase in the number and range of plant-based meat or dairy analogues in the market [3]. Despite their increasing popularity, many consumers still find plant-based foods, especially meat or dairy analogues, to be unsatisfying in terms of flavour [4,5,6,7].

The flavour of a food is perceived via a complex combination of the olfactory (odour/aroma), gustatory (taste), and trigeminal sensations experienced during eating. The volatile organic compounds (VOCs) are released into the mouth and move to olfactory receptors in the nose and non-volatile organic compounds released from the food are sensed by gustatory receptors on the tongue [8,9,10,11]. A large number of VOCs are responsible for the flavour associated with a food [9,10].

While plant-based substrates can contain desirable VOCs, direct extraction is difficult due to their low concentration and recovery rate [12]. Fermentation is a promising way to generate increased concentrations of desired VOCs from plant-based substrates [13,14]. Micro-organisms can metabolise plant substrates and generate secondary volatile metabolites, including desirable meat or dairy flavour notes or precursors [15]. Because of the complex composition of plants, it is challenging to understand what directly influences the production of VOCs. To better understand the specific VOCs produced as compounds present in the plant substrates are altered, a defined medium is essential.

Recent publications have highlighted that lactic acid bacteria (LAB) play a significant role in plant-based fermentations and produce an array of VOCs [16,17]. Defined medium with minimal nutrients have previously been developed to support the growth of LAB [18,19,20,21,22,23]. A carbon source (simple sugars/non-sugar) is a key component of the defined medium as it functions as both an energy source and a flavour precursor [13]. Different carbon sources can influence LAB’s growth and metabolic processes, which results in the production of different VOCs [24,25,26]. The current study investigated the effects of three carbon sources (either glucose, fructose or citrate) on the production of VOCs by LAB in a defined medium.

The selection of LAB strain is another important factor in fermentation studies because the production of VOCs is strongly strain dependent [27]. The obligate heterofermentative LAB, *Levilactobacillus brevis* WLP672 (*Lev. brevis* WLP672), which ferments hexose sugars via the phosphoketolase (PK) pathway [28], was chosen for the current study, as our previous study indicated it grew well in a defined medium and produced a wide range of VOCs [29].

The most commonly used VOC extraction method is headspace solid-phase microextraction (HS-SPME), which provides reasonably high throughput performance without requiring extensive sample preparation [30,31]. Extracted VOCs are commonly detected and analysed by gas chromatography–mass spectrometry (GC-MS) [32]. This technique, while effective, has a number of drawbacks, including a limited mass range and the presence of artifacts in the chromatogram; it is also labour intensive and time consuming [33,34,35]. A more efficient analysis approach is the use of direct injection mass spectrometers (DIMS), which offer quick, direct, and non-invasive VOC identification without the need for prior separation. Proton transfer reaction–mass spectrometry (PTR-MS) is a well-known technique in this class. The fundamental principles of PTR-MS have been well covered in past reviews [36,37]. PTR-MS provides high sensitivity (parts per trillion (ppt) by volume) and real-time VOC monitoring. The main challenge with PTR-MS applications is that identification is based on the molecular formula without the capability to separate isomers. Thus, parallel GC-MS and/or fastGC-PTR-MS analysis is usually required for definitive compound identification [38,39,40].

The current study used PTR-MS coupled with time-of-flight analyser (ToF), HS-SPME-GC-MS, and fastGC-PTR-ToF-MS to determine the VOCs produced by *Lev. brevis* WLP672 growing in a defined medium containing different carbon sources (either glucose, fructose, or citrate).

## 2. Results and Discussion

### 2.1. Physicochemical Properties after Fermentation

*Lev. brevis* WLP672 (thereafter referred to as LB672) grew well in the defined medium, which contained either glucose (DM) or fructose (DMFr) as a carbon source, as indicated by a decrease in pH (due to acid production) [41] and an increase in optical density (OD_600_) values (resulting from cell growth) (Table 1). Notably, the pH reduction was higher after LB672 fermentation in the DM compared to DMFr; however, there were no differences between the individual media at either 25 or 35 °C. In contrast, for OD_600_, the values of DM and DMFr media after fermentation were higher at 25 °C than at 35 °C. The OD_600_ was also higher in DM compared to DMFr. Hence, owing to the differences in OD_600_ at the end of fermentation, temperature and carbon source were assumed to have impacted on the growth of LB672.

There was no growth observed in the defined medium containing citrate as a carbon source (DMCi), as evidenced by no or slight pH reductions and no or small increases in OD_600_ values (Table 1). C4 ketones, such as diacetyl, and acetoin, can be produced from citrate. Previously *Lev. brevis* strains have been reported to produce diacetyl and acetoin from citrate. Citrate metabolism occurs in LAB which possesses citrate permease and lyases activities (citrate-positive LAB) [42]. Only a few of the citrate-positive LAB can grow on citrate when it is the sole carbon source. However, the majority of citrate-positive LAB can co-metabolise citrate when glucose, lactose, or another carbon source is present [43]. From this study, it is not possible to determine if LB672 is a citrate-negative LAB even though growth was not observed. Further research using a defined medium containing citrate and another carbon source (sugar) would be required to better understand the co-metabolism of citrate/sugar by LB672.

As LB672 did not grow in DMCi, the VOCs detected were not included in the following results.

### 2.2. VOCs Produced during Fermentation

LB672, which is an obligatory heterofermentative LAB, ferments sugars via the PK pathway and produces a mixture of primary products such as lactic acid, acetic acid, ethanol, and CO_2_ as well as hundreds of volatile secondary metabolites. These volatile secondary metabolites are responsible for flavour notes or flavour precursors [28,44].

A total of 267 mass peaks (*m*/*z*) were extracted from the raw PTR-ToF-MS data obtained from the headspace of vials containing LB672 growing at either 25 or 35 °C. After the removal of isotopologues and *m*/*z* that were not significantly (*p* > 0.05) different from the baseline data, the number of *m*/*z* was reduced from 267 to a finalised list of a 104 *m*/*z* (Table S1). The tentative identification (t.i.) of each of the 104 *m*/*z* was based on its exact mass, supporting HS-SPME-GC-MS identification for 29 of the 104 (Table 2, Table 3, and Table S1), fastGC-PTR-ToF-MS identification and/or literature data.

The concentrations of all finalised *m*/*z* were highest after 7 days. At 14 days, the concentration of most *m*/*z* decreased as growth stopped and gas flushing removed the VOCs. Therefore, in order to better determine the effects of carbon sources on the VOCs produced, only the data obtained after 0 and 7 days of fermentation were compared.

**Figure 1:**
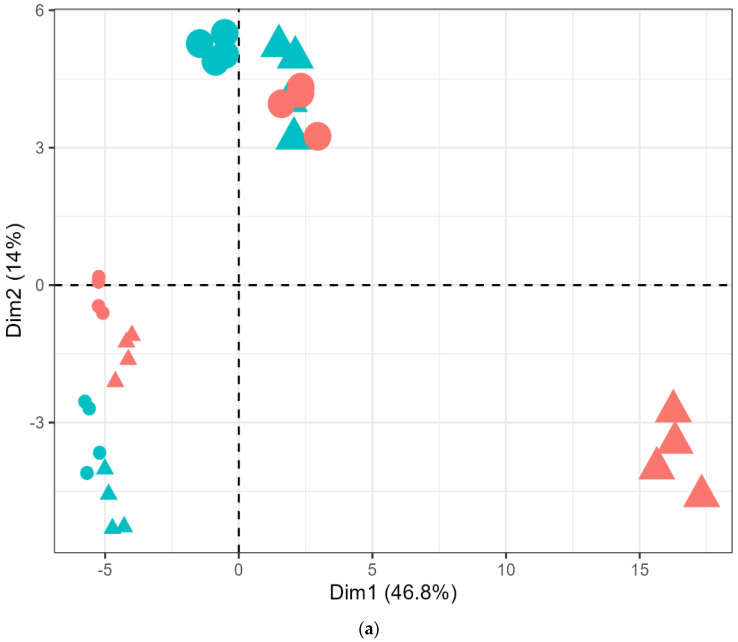

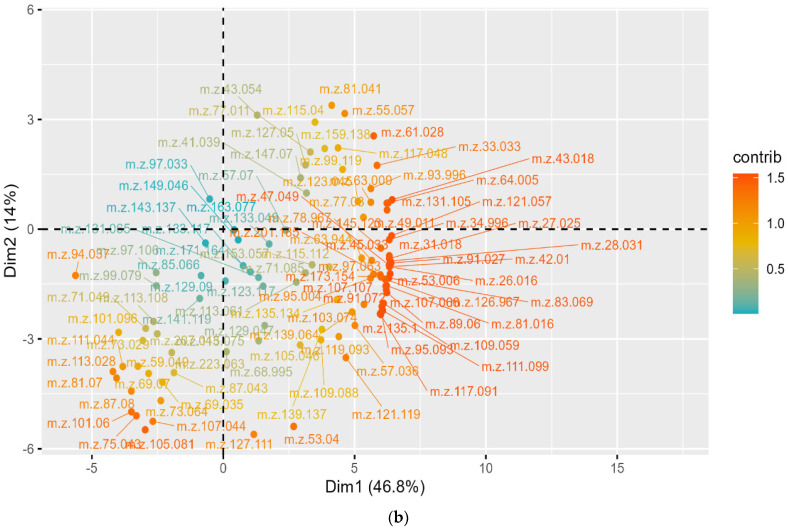
(**a**) Score plot of the principal components (PC) of VOCs produced by LB672 across defined medium compositions (DM: glucose added (red colour), DMFr: fructose added (blue colour)) at 0 (smaller size) and 7 (larger size) days of fermentation at either 25 (symbol-circle) or 35 (symbol-triangle) °C based on the concentrations (ppbV) of finalised 104 *m*/*z* (auto centred and scaled) from PTR-ToF-MS. (**b**) Loadings plot of the principal components (PCs) of VOCs produced by LB672 across defined medium compositions (DM: glucose added, DMFr: fructose added) at 0 and 7 days of fermentation at either 25 or 35 °C based on the concentrations (ppbV) of finalised 104 *m*/*z* (auto centred and scaled) from PTR-ToF-MS.

To understand how glucose or fructose addition to the defined medium and fermentation conditions impacted on VOCs generation during LB672 fermentation, principal component analysis (PCA) was performed using the 104 *m*/*z* detected by PTR-ToF-MS. To ensure that the PCA was focusing on variation in the data from different medium compositions and fermentation conditions, data from the control (uninoculated) treatments were excluded from the PCA. As shown in Figure 1a, the PCA score plot of the DM and DMFr samples explained 60.8% of the total variance, comprising 46.8% from the first principal component (Dim 1) and 14% from the second principal component (Dim 2). The explained variance was mainly attributed to the separation of DM samples at 7 days at 35 °C from the 0 day samples (Figure 1a). The DMFr at either 25 or 35 °C and DM at 25 °C samples showed a minor separation from the 0 day samples. The separation along Dim 1 was attributed to the presence of higher concentrations of *m*/*z* 43.018 (common fragment), 121.057 (t.i. benzeneacetaldehyde), 91.027 (t.i. methyl thiolacetate/mercaptoacetone), 53.006, 28.031, 42.01, 34.996, 26.016 (common fragment), 31.018, 45.033 (t.i. acetaldehyde), 27.025, 47.049 (t.i. ethanol), 83.069, 64.005, 49.011 (t.i. methanethiol), 89.060 (t.i. ethyl acetate), 81.016, 107.066 (t.i. methionol), 107.107, and 131.105 (t.i. isoamyl acetate) associated with DM samples at 7 days at 35 °C, in particular 43.018, 47.049, and 45.033 *m*/*z* (Figure 1b). Dim 2-explained variation was attributed to the separation of 7 days of DMFr at 25 and 35 °C, and DM at 25 °C samples from day 0 samples of DMFr at 25 and 35 °C, and day 7 samples of DM at 35 °C (Figure 1a). The separation along Dim 2 was mainly attributed to *m*/*z* 127.111, 105.081, 53.04, 107.044 (t.i. benzaldehyde), 75.043 (t.i. propanoic acid), 101.06 (t.i. 2-propenyl acetate), 73.064 (t.i. butanal), 87.08 (t.i. 2-methyl butanal and 3-methyl butanal), and 69.035 (t.i. furan) (Figure 1b).

Three-way analysis of variance (ANOVA) was used to determine that of the 104 finalised *m*/*z*, a total number of 66, 82, 74, 71, 55, 68, and 55 *m*/*z* were significantly (*p* < 0.05) differentiated based upon the carbon source (medium composition), time (at 0 and 7 days), temperature (25 and 35 °C), medium composition × time interactions, medium composition × temperature interactions, time × temperature interactions, and medium composition × time × temperature interactions, respectively (Table S1). Finally, 54 *m*/*z* were selected (Table 3 and  Table S2) for which there was a significant (*p* < 0.05) increase in their concentration during fermentation and significant (*p* < 0.05) differences in either main effects or interaction effects.

**Figure 2:**
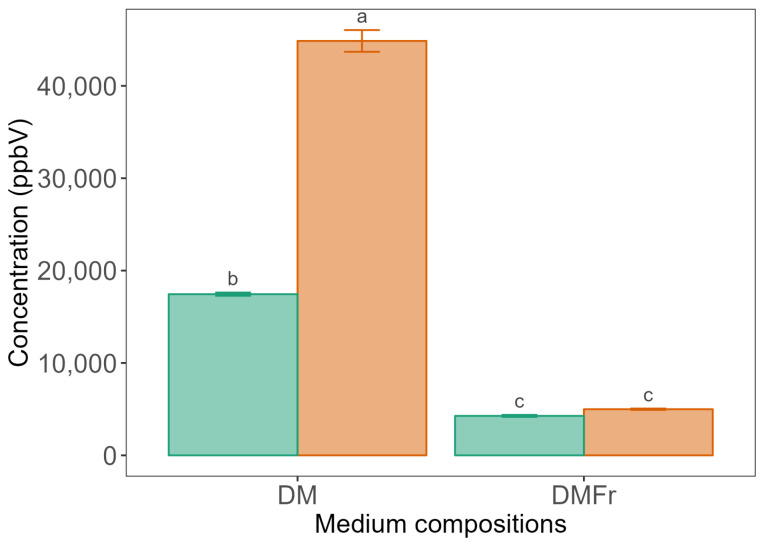
The mean concentration (ppbV) of *m*/*z* 47.049 (t.i ethanol) after 7 days of fermentation by LB672 across defined medium compositions (DM: glucose added, DMFr: fructose added) at either 25 (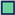) or 35 (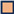) °C. Values are presented as mean ± standard error (*n* = 4). Different superscript lowercase letters represent significant differences between the carbon sources at either 25 or 35 °C according to Tukey’s test at *p* < 0.05.

**Figure 3:**
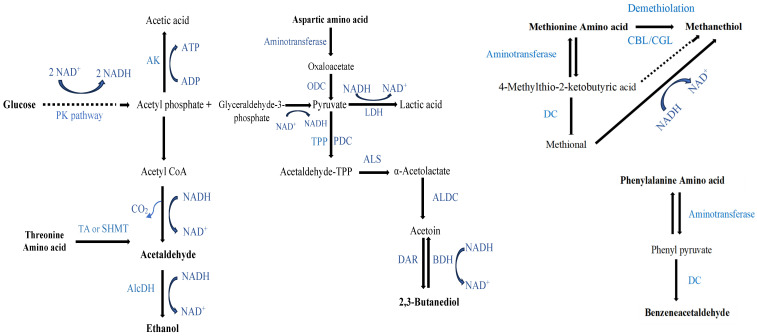
The proposed metabolic pathways of LB672 fermentation in the defined medium contained glucose and an amino acid mixture (DM). Dotted lines indicate a series of enzymatic or chemical reactions. Adapted from Bamforth and Cook [15], Quintans et al. [43], Fernandez and Zuniga [46], Christensen et al. [47], Ardö [48], Marilley and Casey [54], Laëtitia et al. [56], Le Bars and Yvon [57]. PK: Phosphoketolase, AlcDH: Alcohol dehydrogenase, LDH: Lactate dehydrogenase, ODC: Oxaloacetate decarboxylase, AK: Acetokinase, TA: threonine aldolase, SHMT: serine hydroxymethyltransferase, PDC: Pyruvate decarboxylase, TPP: Thiamine pyrophosphate, ALS: α-Acetolactate synthase, ALDC: α-Acetolactate decarboxylase, DAR: Diacetyl acetoin reductase, BDH: 2,3-butanediol dehydrogenase, CBL: cystathionine β-lyase, CGL: cystathionine γ-lyase, and DC: decarboxylase.

Ethanol, which is a key marker compound in fermentation studies, is an end product of sugar fermentation by heterofermentative LAB [15]. After 7 days of LB672 fermentation, the concentration of *m*/*z* 47.049 (t.i. ethanol) (Figure 2) was significantly (*p* < 0.05) higher in DM at either 25 or 35 °C than in DMFr at either 25 or 35 °C. In DM, the concentration of ethanol was significantly (*p* < 0.05) higher at 35 °C compared to 25 °C. When glucose is fermented by *Lev. brevis* via the PK pathway, an extra two NAD(P)H are released during conversion of the glucose to a five-carbon sugar. The extra NAD(P)H produced is transferred to acetyl-CoA, yielding ethanol (Figure 3). In the presence of external electron acceptors like fructose, the extra NAD(P)H is transferred to the acceptors, resulting in the generation of large amounts of mannitol, which is not reduced to ethanol. When fructose is present as the only sugar, it serves both as a substrate for the PK pathway and as an electron acceptor [45], which could account for the low ethanol concentration detected in DMFr. It has previously been reported that the concentration of ethanol was high in *Limosilactobacillus fermentum* (*Lim. fermentum*) growing in medium with added glucose, whereas no ethanol was detected in medium with added fructose [24].

Ethanol can also be produced by LAB through the degradation of the amino acid (AA), threonine (Thr). Thr catabolism produces acetaldehyde using either threonine aldolase (TA) or serine hydroxymethyltransferase (SHMT) enzymes, with the acetaldehyde being converted to ethanol in the presence of the enzyme, alcohol dehydrogenase (AlcDH) [46,47,48] (Figure 3). While LB672 may or may not have a *glyA* gene-encoding TA and SHMT enzymes, the Thr present in the defined medium was 50 times lower than the glucose concentration, suggesting that glucose metabolism rather than Thr catabolism was most likely the source of ethanol detected.

**Figure 4:**
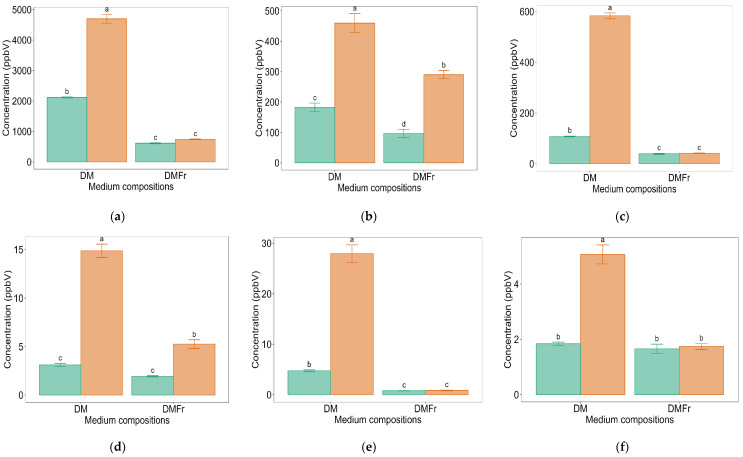
Mean concentrations (ppbV) of *m*/*z* 45.033 (t.i. acetaldehyde) (**a**) *m*/*z* 49.011 (t.i. methanethiol) (**b**) *m*/*z* 89.060 (t.i. ethyl acetate) (**c**) *m*/*z* 91.027 (t.i. methyl thiolacetate/mercaptoacetone) (**d**) *m*/*z* 91.072 (t.i. 2,3 butanediol) (**e**) and *m*/*z* 121.057 (t.i. benzeneacetaldehyde) (**f**) across defined medium compositions (DM: glucose added, DMFr: fructose added) at 25 (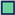) and 35 (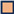) °C after 7 days of fermentation by LB672. Values are presented as mean ± standard error (*n* = 4). Different superscript lowercase letters represent significant difference between the carbon sources at either 25 or 35 °C according to Tukey’s test at *p* < 0.05.

The concentration of *m*/*z* 45.033 (t.i. acetaldehyde) after 7 days of LB672 fermentation was significantly (*p* < 0.05) higher in DM at either 25 or 35 °C than in DMFr at either 25 or 35 °C (Figure 4a). In DM, the concentration of acetaldehyde was significantly (*p* < 0.05) higher at 35 °C than 25 °C. Acetaldehyde, which is one of the major flavour compounds in yoghurt [49], is produced by LAB either from sugars via the PK pathway [15] or from Thr catabolism using TA or SHMT enzymes [46,48] (Figure 3). Even though the defined media DM and DMFr used in this study contained Thr at the same proportion, the concentration of acetaldehyde varied significantly (*p* < 0.05) based on whether glucose or fructose was in the medium. Given the differences in the concentration of sugar (carbon source) and Thr in the defined medium, it is speculated that the acetaldehyde produced was from mainly sugar metabolism. To confirm this, further research using the same experimental design in the defined medium (DM/DMFr) with and without the addition of Thr is required. Acetaldehyde improves desirable yoghurt flavour when present at greater than 8 mg/kg; however, at higher concentrations (200 mg/kg or above), it may negatively influence the overall yoghurt flavour [44,50]. In the current study, the highest acetaldehyde concentration after LB672 fermentation in the DM was well below the concentration that has been reported to adversely affect flavour. Note that the acetaldehyde concentration is the headspace concentration, not the total concentration in the fermented sample. Further, it is important to note that the concentration of the specific VOC, its perception limit, and presence of other VOCs are crucial factors when considering the desired flavour notes.

The concentration of *m*/*z* 89.060 (t.i. ethyl acetate) followed a similar pattern to acetaldehyde (Figure 4c). The ester, ethyl acetate, which is formed by alcohol acetyltransferases from the reaction between acetyl Co-A and ethanol, can confer fruity notes [41]. It is obvious that the production of ethyl acetate varied significantly (*p* < 0.05) based on whether glucose (DM) or fructose (DMFr) was present in the defined medium. The signal for *m*/*z* 89.060 was considered to be mainly ethyl acetate, with a minor contribution from ethyl butanoate but not butyric acid, 3-methyl butanol, or acetoin. These contributions were based on the absence of acetoin detected in HS-SPME-GC-MS analysis (Table 2) or in fastGC-PTR-ToF-MS. FastGC-PTR-ToF-MS detected 3-methyl butanol in samples, where the signal was detected at *m*/*z* 71.085 (main fragment) but not at 89.060 (Table 3) and confirmed with authentic standard. Further, based on the retention time (RT) of main and fragment ions detected in fastGC-PTR-ToF-MS (Table 4), the *m*/*z* 89.060 detected by PTR-ToF-MS was not butyric acid, though a small signal was observed for ethyl butanoate.

The concentration of *m*/*z* 49.011 (t.i. methanethiol) was significantly (*p* < 0.05) higher in DM at 35 °C than in all other treatments after 7 days of fermentation. In addition, methanethiol in DMFr at 35°C was significantly higher (*p* < 0.05) than in DM and DMFr at 25 °C (Figure 4b). Methanethiol, which is an odour-active flavour compounds in meat [51] and cheese [52], can be produced by LAB from methionine (Met), a sulphur-containing AA, through either a transamination reaction, demethiolation, or through methional using various enzymes [46,53,54,55] (Figure 3). Even though Met was present in the two defined media (DM and DMFr) used at the same proportion, the concentration of methanethiol was higher in the glucose-containing medium compared to the fructose-containing medium. This difference could be because the extra NADH generated during the metabolism of glucose by LB672 via the PK pathway is used in the methanethiol production pathway (Figure 3). Furthermore, the concentration of *m*/*z* 91.027 (t.i. methyl thiolacetate/ mercaptoacetone) followed a similar pattern to methanethiol (Figure 4d).

The concentration of *m*/*z* 91.072 (t.i. 2,3-butanediol) was significantly (*p* < 0.05) higher in DM at either 25 or 35 °C compared to DMFr at either 25 or 35 °C (Figure 4e). 2,3-butanediol can be produced from pyruvate which is produced either from sugar metabolism or aspartic acid (Asp) catabolism through an intermediate acetoin using the diacetyl acetoin reductase enzyme (DAR) (Figure 3) [43,56,57]. Even though the defined media DM and DMFr contained Asp at the same proportion, the concentration of *m*/*z* 91.072 varied significantly (*p* < 0.05) based on whether glucose or fructose was present in the medium. As a result, it is speculated that the *m*/*z* 91.072 produced in the current study mainly resulted from pyruvate, which is produced from glucose metabolism. To confirm this, further research using the same experimental design in the defined medium (DM/DMFr) with and without the addition of Asp is required. Once 2,3 butanediol is produced, it can subsequently be converted to acetoin by the 2,3-butanediol dehydrogenase enzyme (BDH) (Figure 3). As acetoin was not detected in this study, it is speculated that DAR but not BDH is present in LB672.

The concentration of *m*/*z* 121.057 (t.i. 2,3 benzeneacetaldehyde) was significantly (*p* < 0.05) higher in DM at 35 °C compared to all other treatments (Figure 4f). Benzeneacetaldehyde, which is one of the key flavour compounds in cheese [52], can be produced by LAB from phenylalanine through a transamination reaction, followed by decarboxylation reaction (Figure 3) [46].

Overall, the glucose-containing defined medium (DM) showed higher concentrations of VOCs (*m*/*z*) of interest after fermentation by LB672 compared to the fructose-containing defined medium (DMFr) at either 25 or 35 °C. Among the detected fermented VOCs in the DM, acetaldehyde, methanethiol, and benzeneacetaldehyde are key VOCs in dairy products [49,52], and methanethiol is a key VOC in meat products [51].

Though LB672 grew well in the defined medium DMFr, the concentrations of VOCs detected in this study were low. It has previously been reported that mannitol (sugar alcohol) was the main compound produced by *Lim. fermentum* growing in the medium containing fructose, whereas ethanol was the main compound in the medium containing glucose [24]. Note, however, that the low concentration of ethanol produced in the defined medium DMFr, in the current study, could be a benefit for developing low-ethanol containing plant-based fermented foods or ingredients.

It was of interest that the concentrations of specific VOCs were higher at 35 °C compared to 25 °C, despite the actual growth of LB672, based on OD_600_ values being higher at 25 °C compared to 35 °C. This observation suggests that temperature influenced the metabolic processes of LAB, which in turn impacted on the production of VOCs.

It is important to appreciate that many other compounds in the defined medium are likely to impact on VOC production [13,58,59,60,61,62]. Therefore, the production of specific VOCs that mimic meat or dairy flavours by LB672 will be better understood by examining other medium compositions in the defined medium using a similar experimental design.

## 3. Materials and Methods

### 3.1. LAB Strain

LB672 was supplied by White Labs, USA and kept at 4 °C prior to use. For activation, 1 mL of the stock culture was taken and added to 10 mL of de Man, Rogosa, and Sharpe (MRS) broth, which was incubated at 25 °C for 3 days in sealed containers using anaerobic packs (Mitsubishi Gas Chemical (MGC) Company, Tokyo, Japan). An aliquot of the resulting culture was inoculated onto MRS agar medium using the streak plate method to obtain single colonies and incubated at 25 °C for 3 days using MGC anaerobic packs. An inoculating suspension was prepared by adding colonies from the streak plate to 10 mL of MRS broth, which was incubated at 25 °C for 3 days using MGC anaerobic packs. Cells were pelleted by centrifugation (5000× *g* for 5 min at 20 °C) (PK 121R/ALC International, Cologno Monzese, Italy) and washed twice with sterilised phosphate-buffered saline (PBS) (100 mL; 0.8 g NaCl, 0.02 g KCl, 0.144 g Na_2_HPO_4_, and 0.0245 g KH_2_PO_4_, pH of 7.4) and then resuspended to a final concentration of 1 × 10^9^ CFU/mL. The resulting suspension was used as inoculum in the fermentation trials.

### 3.2. Medium Composition

The defined medium used in this study was based on earlier research [18,22,23,63,64,65,66] and refined through trials. The defined medium contained the following ingredients: D-glucose (20 g/L), peptone (Bacto peptone, enzymatic protein digest) (5 g/L), sodium acetate (12 g/L), mineral salts (MgSO_4_·7H_2_O, (0.2 g/L) NaCl (0.01 g/L), FeSO_4_·7H_2_O (0.01 g/L), and MnSO_4_·5H_2_O (0.04 g/L), and vitamins (calcium pantothenate (B5) (0.4 mg/L), nicotinic acid (B3) (0.2 mg/L), riboflavin (B2) (0.4 mg/L), and thiamine HCl (B1) (0.2 mg/L)), and an amino acid mixture (0.4 g/L of each amino acids; l-leucine, l-isoleucine, l-phenylalanine, l-glutamic acid, l-aspartic acid, l-threonine, or l-methionine). Three types of defined media were prepared as follows: 1. the original defined medium (glucose as a carbon source; DM), 2. the defined medium with fructose (glucose replaced by fructose (20 g/L); DMFr), and 3. the defined medium with sodium citrate (glucose replaced by sodium citrate (20 g/L); DMCi). The amino acids were dissolved in HCl solution (50 mM). All stock solutions were prepared using deionised water unless otherwise stated. The glucose and vitamin solutions were filter sterilised using a 0.22 µm syringe filter (Nylon membrane; BIOFIL, Kowloon, Hong Kong), and all other components were sterilised by autoclaving at 121 °C for 15 min. Unless otherwise specified, all of the chemicals used were of analytical grade. All procedures were carried out in a Class II biological safety cabinet.

### 3.3. Fermentation

Prior to fermentation, the prepared defined media were held for 3 days at 25 °C to ensure sterility. Then, 4 mL aliquots of defined media were transferred into sterile headspace vials (20 mL) capped with PTFE/silicone septa (Agilent, Cernusco sul Naviglio, Italy). A 0.05 mL aliquot of the LB672 cell suspension (1 × 10^9^ CFU/mL) was inoculated to each headspace vial, which were flushed with N_2_ at a rate of 10 mL/min for 20 min to establish an anaerobic environment. The vials were placed in sample trays in a randomised order in an autosampler (MPS Multi-Purpose Sampler, Gerstel, Germany) and held at either 25 or 35 °C for 14 days. Eight replicates were prepared from each sample, four of which were kept at either 25 °C or 35 °C. Controls were uninoculated defined media. At the end of the fermentation (after 14 days), growth was confirmed by measuring the pH (inoLab Level 1/WTW, Weilheim, Germany) and optical density (BioPhotometer/Eppendorf, Hamburg, Germany) of a sub-sample of the fermented culture.

### 3.4. Determination of VOCs 

#### 3.4.1. PTR-ToF-MS

The VOCs produced during fermentation were measured at three time points (0, 7, and 14 days of fermentation) using a PTR-ToF-MS 8000 (Ionicon Analytik GmbH, Innsbruck, Austria). The drift tube conditions were as follows: 110 °C drift tube temperature, 2.8 mbar drift pressure, 628 V drift voltage. This led to an E/N ratio of about 140 Townsend (Td), with E corresponding to the electric field strength and N to the gas number density (1 Td = 10^–17^ V cm^2^). The sampling time per channel of ToF acquisition was 0.1 ns, amounting to 350,000 channels for a mass spectrum ranging up to *m*/*z* = 340, which resulted in the acquisition rate of 1 spectrum/s. Each measurement was conducted automatically using a multipurpose GC automatic sampler (Gerstel GmbH, Mulheim am Ruhr, Germany), and 60 s between each measurement was applied to prevent the memory effects/carry over. The sample headspace was withdrawn with the 2.5 mL syringe (CTC Analytics AG, Zwingen, Switzerland) and injected into the static headspace (SHS) module (Ionicon Analytik GmbH, Innsbruck, Austria). The flow of zero air inside the static headspace module was 90 sccm, and the syringe was injected with the speed 100 µL/s, which provoked a 16-fold dilution of the sample. The injection time was 25 s/sample [67]. Pure N_2_ was flushed through the syringe immediately before withdrawal to prevent the contamination of a measurement. PTR-MS performances were verified with certified calibration mixtures. Sensitivity was better than 10 cps/ppbv, and the limit of detection (LOD) was lower than 100 pptv at an acquisition rate of 1 spectrum/s. Mass resolution was better than 4000 M/ΔM. The internal mass axis calibration of mass spectral data, and peak extraction were performed according to previously described procedures [68,69]. The peak intensity in ppb/v (parts per billion by volume) was estimated using the formula described in the literature. The formula uses a constant value for the reaction rate coefficient (k = 2·10^−9^ cm^3^ s^−1^) [70].

#### 3.4.2. HS-SPME-GC-MS

HS-SPME-GC-MS measurements were included to help with identification of compounds detected by PTR-ToF-MS. At the end of fermentation (after 14 days), samples were removed from the PTR-ToF-MS autosampler sample tray and transferred to a GC-MS autosampler sample tray held at either 25 or 35 °C. A SPME fibre (Divinylbenzene/Carboxen/Polydimethylsiloxane (DVB/CAR/PDMS) 2 cm, 50/30 µm thickness) was exposed to the headspace of the sample for 40 min at either 25 or 35 °C. VOCs were desorbed from the SPME fibre at 250 °C for 5 min in the injector of the GC in splitless mode, and helium was used as the carrier gas at a flow rate of 2 mL/min. Volatiles were separated using a capillary column (InnoWax 30 m/0.32 mm/0.5 µm). The oven temperature program was set 40 °C held for 1 min, and then increased to 250 °C at 5 °C/min and held for 2 min. MS was performed with an ion source temperature of 200 °C and an electron ionisation energy of 70 eV over the mass range of *m*/*z* 33–350.

According to the retention time (RT) of n-alkane series (C7-C30) obtained under the same conditions, retention indices (RI) of the detected VOCs were computed. By comparing RI calculated and from the NIST library (NIST14, version 2.2, National Institute of Standards and Technology), the VOCs were identified.

#### 3.4.3. FastGC-PTR-ToF-MS

To assist with attributing each signal (*m*/*z*) to the correct compound and determining the number of compounds contributing to each *m*/*z* (isomers), fastGC-PTR-ToF-MS was carried out on all samples at each time point after performing SHS-PTR-ToF-MS measurements. The drift tube conditions were same as described in Section 3.4.1. The polar capillary column (MXT^®^-WAX (Siltek^®^—treated stainless steel), 6 m) was maintained under pure helium with a constant flow rate of 2.5 sccm. Pure N_2_ was used as a make-up gas with a flow of 50 sccm. Sample headspace air was injected into fastGC sampling loop for 15 s to ensure complete filling. The chromatographic measurement was registered for 250 s with the thermal ramp from 40 to 200 °C and data acquisition was set to 5 spectra/s [71]. The following pure standards, ethyl acetate, ethyl butanoate, ethyl hexanoate, ethyl octanoate, ethyl decanoate, ethanol, 2-methyl propanol, 3-methyl butanol, phenylethyl alcohol, 2-butanone, 2-hexanone, 2-heptanone, 2-nonanone, and benzaldehyde were prepared individually and diluted to a final concentration of 10 ppm through serial dilutions. Acetic acid was diluted to a final concentration of 50 ppm through serial dilutions. The pure standards were also analysed in fastGC-PTR-ToF-MS to improve the confidence of each *m*/*z* identified. TofDAQViewer was used to visually inspect fastGC-PTR-ToF-MS data for the standards and samples after they were saved as h5-files. From the standards, a table consisting of RT, literature RI, and fragmentation pattern, which in combination with literature fragmentation patterns and GC-MS results, was used to assign compound identities to each *m*/*z* (Table 5).

### 3.5. Statistical Analysis

To determine which sample signals (*m*/*z*) were significantly (*p* < 0.05) higher than detected in the blanks, a series of ANOVA was run between the blanks and each sample type.

PCA was performed on all samples using all sample-related *m*/*z* concentrations and coded to highlight the sample differentiation based on medium carbon source, fermentation temperature and fermentation time in R (version 4.2.1, R Foundation for Statistical Computing, Vienna, Austria) using “factoextra”, “ggplot2”, “reshape”, “ggpubr”, and “dplyr” packages [72]. Data were normalised by autoscaling (mean-centred and divided by the standard deviation of each variable) using the “prcomp” function of the “factoextra” package.

To identify significant *m*/*z*, all sample-related *m*/*z* were subjected to three-way ANOVA using a general linear model (significance level at *p* < 0.05), where the main effects were medium carbon source, fermentation temperature and fermentation time and all interactions were investigated. The mean separations for each *m*/*z* were calculated using Tukey’s HSD test at *p* < 0.05. Analysis was carried out using SPSS (IBM SPSS statistics, version 29.0.0.0 (241), USA).

Selected VOCs (*m*/*z*) were plotted in bar graphs for main factors of medium carbon source, and fermentation temperature as well as their interaction effects at 7 days of fermentation using “ggplot2”, “dplyr”, “ggpubr”, “reshape”, “ggthemes”, “multcompView”, “readr”, and “scales” packages in R. The mean separations for each *m*/*z* were calculated using Tukey’s HSD test at *p* < 0.05.

## 4. Conclusions

The VOCs produced by LB672 growing in a defined medium containing either glucose or fructose as a carbon source under different fermentation conditions were assessed using PTR-ToF-MS, and the compounds detected were confirmed using HS-SPME-GC-MS, fastGC-PTR-ToF-MS, and/or the literature data. For the first time, PTR-ToF-MS was used to track the generation of fermentation VOCs in a defined medium. VOC production by LB672 was strongly influenced by the carbon source and fermentation time and temperature. The VOCs detected were mainly acids, alcohols, aldehydes, esters, furans, ketones, and sulphur compounds. Overall, the defined medium containing glucose (DM) generated higher concentrations of the VOCs of interest after 7 days of fermentation at 35 °C compared to at 25 °C and fructose-containing defined medium (DMFr) at both temperature conditions. This study suggests that the production of target VOCs in plant-based fermentations may be enhanced by altering the carbon source or fermentation conditions. This knowledge could be applied to the production of target VOC, on an industrial scale, through the fermentation of plant-based substrates.

## Figures and Tables

**Table 1:** The mean pH and OD_600_ of the samples after 14 days of LB672 fermentation in different medium compositions. Values are the mean ± standard error of two replicates. Values with different superscript lowercase letters (^a–b^) in the row (either pH or OD_600_) are significantly different according to Tukey’s test at *p*< 0.05.

Medium	Initial pH	at 25 °C		at 35 °C	
		pH	OD_600_	pH	OD_600_
DM	5.67 ± 0.035 ^a^	4.47 ± 0.011 ^b^	1.40 ± 0.005 ^a^	4.44 ± 0.1 ^b^	1.20 ± 0.005 ^b^
DMFr	5.68 ± 0.025 ^a^	4.72 ± 0.03 ^b^	1.26 ± 0.06 ^a^	4.65 ± 0.04 ^b^	1.06 ± 0.04 ^b^
DMCi	6.27 ± 0.005 ^a^	6.27 ± 0.0045 ^a^	0.07 ± 0.005 ^a^	6.27 ± 0.0005 ^a^	0.05 ± 0.0025 ^a^

DM: original defined medium (glucose); DMFr: defined medium with fructose; DMCi: defined medium with citrate.

**Table 2:** VOCs detected after 14 days of LB672 fermentation in DM with either added glucose or fructose at either 25 or 35 °C by HS-SPME-GC-MS.

No	VOCs	Formula	RT	RI.cal	RI.lit
	Acids				
1	Acetic acid	C_2_H_4_O_2_	15.29	1467	1449
2	Butyric acid	C_4_H_8_O_2_	19.626	1646	1625
3	Hexanoic acid	C_6_H_12_O_2_	24.439	1862	1846
4	Octanoic acid	C_8_H_16_O_2_	28.755	2035	2060
5	Decanoic acid	C_10_H_20_O_2_	32.698	2154	2276
	Alcohols				
6	2-Propanol	C_3_H_8_O	3.068	934	927
7	Ethanol	C_2_H_6_O	3.162	941	932
8	2-Pentanol	C_5_H_12_O	6.687	1134	1119
9	1-Butanol	C_4_H_10_O	7.271	1158	1142
10	2/3-Methyl-1-butanol	C_5_H_12_O	8.862	1220	1208/1209
11	3-Methyl-3-buten-1-ol	C_5_H_10_O	9.99	1263	1248
12	2-Heptanol	C_7_H_16_O	11.784	1332	1320
13	Hexanol	C_6_H_14_O	12.671	1365	1355
14	2,3-Butanediol	C_4_H_10_O_2_	17.438	1554	1543
15	1-Octanol	C_8_H_18_O	17.858	1571	1557
16	Menthol	C_10_H_20_O	19.806	1653	1637
17	2-Decen-1-ol	C_10_H_20_O	23.788	1832	1806
18	Benzyl alcohol	C_7_H_8_O	25.143	1895	1870
19	Phenylethyl alcohol	C_8_H_10_O	25.853	1930	1906
20	2-Tridecanol	C_13_H_28_O	25.9	1933	1903
21	P-cresol	C_7_H_8_O	29.452	2051	2080
	Aldehydes				
22	Butanal	C_4_H_8_O	2.752	911	877
23	2-Methyl butanal	C_5_H_10_O	2.898	922	914
24	3-Methyl butanal	C_5_H_10_O	2.958	926	918
25	2-Methyl-2-butenal	C_5_H_8_O	6.174	1114	1095
26	3-Methyl-2-butenal	C_5_H_8_O	8.765	1216	1215
27	2-Methyl pentanal	C_6_H_12_O	13.655	1403	-
28	Benzaldehyde	C_7_H_6_O	17.154	1542	1520
29	Benzeneacetaldehyde	C_8_H_8_O	20.033	1663	1640
	Esters				
30	Ethyl acetate	C_4_H_8_O_2_	2.608	901	888
31	Isoamyl acetate	C_7_H_14_O_2_	6.811	1139	1122
32	2-Phenylethyl acetate	C_10_H_12_O_2_	23.892	1836	1813
	Furans				
33	Furfural	C_5_H_4_O_2_	15.723	1484	1461
34	2-Furanmethanol	C_5_H_6_O_2_	20.403	1679	1660
	Ketones				
35	Acetone	C_3_H_6_O	1.971	823	819
36	2-Heptanone	C_7_H_14_O	8.285	1198	1182
	Sulphur compounds				
37	Dimethyl disulfide	C_2_H_6_S_2_	5.727	1095	1077
38	Methional	C_4_H_8_OS	15.467	1474	1454
39	Cyclohexyl isothiocyanate	C_7_H_11_NS	20.607	1687	1667
40	3-(methylthio)-1-propanol (methionol)	C_4_H_10_OS	21.644	1734	1719
	Pyrazine				
41	Pyrazine	C_4_H_4_N_2_	9.076	1228	1212
	Unknown compounds				
42	Unknown 1		6.04		
43	Unknown 2		12.528		

Retention time (RT). Retention indices (RI).

**Table 3:** The selected VOCS (*m*/*z*) detected by PTR-ToF-MS after LB672 fermentation that significantly (*p* < 0.05) distinguished between defined medium compositions (M), fermentation time (0 and 7 days) (T) and temperature (either at 25 or 35 °C) (Temp), and their interaction effects.

No	*m*/*z*	Sum Formula (Protonated Ion)	Tentative Identification	*p*-Value						
				M	T	Temp	M × T	M × Temp	T × Temp	M × T × Temp
1	26.016	C_2_H_2_^+^	Common fragment	<0.0001	<0.0001	<0.0001	<0.0001	<0.0001	<0.0001	<0.0001
2	27.025	C_2_H_3_^+^		<0.0001	<0.0001	<0.0001	<0.0001	<0.0001	<0.0001	<0.0001
3	28.031	C_2_H_4_^+^		<0.0001	<0.0001	<0.0001	<0.0001	<0.0001	<0.0001	<0.0001
4	31.018	CH_2_OH^+^	Formaldehyde	<0.0001	<0.0001	<0.0001	<0.0001	<0.0001	<0.0001	<0.0001
5	33.033	CH_4_OH^+^		0.969	<0.0001	<0.0001	0.005	0.001	<0.0001	0.003
6	34.996	H_2_SH^+^	Hydrogen sulfide	<0.0001	<0.0001	<0.0001	<0.0001	0.008	<0.0001	0.012
7	41.039	C_3_H_5_^+^		<0.0001	<0.0001	<0.0001	<0.0001	0.005	<0.0001	<0.0001
8	42.010			<0.0001	<0.0001	<0.0001	<0.0001	<0.0001	<0.0001	<0.0001
9	43.018	C_2_H_3_O^+^	Common fragment	<0.0001	<0.0001	<0.0001	<0.0001	<0.0001	<0.0001	<0.0001
10	43.054	C_3_H_7_^+^	Propanol fragment ^1^	<0.0001	<0.0001	<0.0001	<0.0001	<0.0001	<0.0001	<0.0001
11	45.033	C_2_H_4_OH^+^	Acetaldehyde	<0.0001	<0.0001	<0.0001	<0.0001	<0.0001	<0.0001	<0.0001
12	47.049	C_2_H_6_OH^+^	Ethanol ^1^	<0.0001	<0.0001	<0.0001	<0.0001	<0.0001	<0.0001	<0.0001
13	49.011	CH_4_SH^+^	Methanethiol	<0.0001	<0.0001	<0.0001	<0.0001	0.037	<0.0001	0.057
14	53.006			<0.0001	<0.0001	<0.0001	<0.0001	<0.0001	<0.0001	<0.0001
15	55.057	C_4_H_7_^+^		0.986	<0.0001	0.276	0.009	0.723	0.422	0.668
16	57.036	C_3_H_4_OH^+^		0.885	<0.0001	<0.0001	<0.0001	<0.0001	<0.0001	<0.0001
17	57.070	C_4_H_9_^+^	1-Butanol fragment ^1^	<0.0001	<0.0001	<0.0001	0.242	<0.0001	<0.0001	0.066
18	61.028	C_2_H_4_O_2_H^+^	Acetic acid ^1,2,3^	0.324	<0.0001	<0.0001	0.036	<0.0001	<0.0001	0.001
19	63.009	CO_2_*H_3_O^+^		<0.0001	<0.0001	0.190	<0.0001	0.122	0.200	0.128
20	63.944			0.082	<0.0001	<0.0001	0.144	0.200	<0.0001	0.426
21	64.005			<0.0001	<0.0001	<0.0001	<0.0001	0.032	<0.0001	0.062
22	71.085	C_5_H_11_^+^	3-Methyl-butanol fragment ^1,2^	<0.0001	<0.0001	<0.0001	0.998	0.183	0.004	0.540
23	77.011			0.002	<0.0001	0.001	0.045	0.425	<0.0001	0.905
24	77.030			0.004	<0.0001	0.196	0.051	0.009	0.465	0.005
25	78.967	CH_2_S_2_H^+^		0.006	<0.0001	<0.0001	0.019	0.090	<0.0001	0.148
26	81.016			<0.0001	<0.0001	<0.0001	<0.0001	<0.0001	<0.0001	<0.0001
27	81.041	C_4_H_4_N_2_H^+^	Pyrazine^1^	0.046	<0.0001	<0.0001	0.036	0.566	0.732	0.868
28	83.069			<0.0001	<0.0001	<0.0001	<0.0001	<0.0001	<0.0001	<0.0001
29	89.060	C_4_H_8_O_2_H^+^	Ethyl acetate ^1,2,4^	<0.0001	<0.0001	<0.0001	<0.0001	<0.0001	<0.0001	<0.0001
30	91.027	C_3_H_6_OSH^+^	Methyl thiolacetate/Mercaptoacetone	<0.0001	<0.0001	<0.0001	<0.0001	<0.0001	<0.0001	<0.0001
31	91.072	C_4_H_10_O_2_H^+^	2,3-Butanediol ^1^	<0.0001	<0.0001	<0.0001	<0.0001	<0.0001	<0.0001	<0.0001
32	93.996			<0.0001	<0.0001	<0.0001	0.079	0.365	<0.0001	0.365
33	95.004	C_2_H_6_S_2_H^+^	Dimethyl disulfide ^1^	0.004	<0.0001	<0.0001	0.010	0.031	<0.0001	0.050
34	95.093			<0.0001	<0.0001	<0.0001	<0.0001	<0.0001	<0.0001	<0.0001
35	97.063	C_6_H_8_OH^+^	2,5-Dimethylfuran/Cyclohexen-2-one	<0.0001	<0.0001	<0.0001	0.144	<0.0001	<0.0001	<0.0001
36	99.119	C_7_H_15_^+^	2-Heptanol fragment ^1^	0.059	<0.0001	<0.0001	0.076	0.700	<0.0001	0.447
37	103.074	C_5_H_10_O_2_H^+^	C5 esters and acids (pentanoic acid/3-methyl butanoic acid)	0.027	<0.0001	<0.0001	<0.0001	0.017	0.544	0.002
38	107.066	C_4_H_10_OSH^+^	Methionol ^1^	<0.0001	<0.0001	<0.0001	<0.0001	<0.0001	<0.0001	<0.0001
39	107.107			<0.0001	<0.0001	<0.0001	<0.0001	<0.0001	<0.0001	<0.0001
40	109.059	C_7_H_8_OH^+^	Benzyl alcohol ^1^	<0.0001	<0.0001	<0.0001	<0.0001	<0.0001	<0.0001	<0.0001
41	111.099			<0.0001	<0.0001	<0.0001	<0.0001	<0.0001	<0.0001	<0.0001
42	115.112	C_7_H_14_OH^+^	2-Heptanone ^1,2^	0.916	0.009	0.004	0.069	0.717	0.023	0.256
43	117.091	C_6_H_12_O_2_H^+^	Hexanoic acid ^1^	<0.0001	<0.0001	<0.0001	<0.0001	<0.0001	<0.0001	<0.0001
44	121.057	C_8_H_8_OH^+^	Benzeneacetaldehyde ^1^	<0.0001	<0.0001	<0.0001	<0.0001	<0.0001	<0.0001	<0.0001
45	121.119			<0.0001	0.070	<0.0001	<0.0001	<0.0001	<0.0001	<0.0001
46	123.045	C_7_H_6_O_2_H^+^	Benzoic acid	0.006	<0.0001	0.201	0.002	0.004	0.257	0.005
47	126.967			<0.0001	<0.0001	<0.0001	<0.0001	0.004	<0.0001	0.004
48	131.105	C_7_H_14_O_2_H^+^	Isoamyl acetate ^1^	<0.0001	<0.0001	<0.0001	<0.0001	<0.0001	<0.0001	<0.0001
49	135.100	C_6_H_14_O_3_H^+^		<0.0001	<0.0001	<0.0001	<0.0001	<0.0001	<0.0001	<0.0001
50	135.134			0.315	0.033	<0.0001	0.044	0.285	0.012	0.250
51	139.064			<0.0001	0.009	0.005	0.002	<0.0001	0.005	0.001
52	145.123	C_8_H_16_O_2_H^+^	Octanoic acid ^1^	0.001	<0.0001	0.001	0.003	0.018	0.022	0.006
53	173.154	C_10_H_20_O_2_H^+^	Decanoic acid ^1^	<0.0001	<0.0001	0.001	<0.0001	0.003	<0.0001	<0.0001
54	201.185	C_12_H_24_O_2_H^+^	Decanoic acid ethyl ester	<0.0001	<0.0001	<0.0001	<0.0001	<0.0001	0.001	<0.0001

1: *m*/*z* identified by HS-SPME-GC-MS. 2: *m*/*z* identified by fastGC-PTR-ToF-MS + injection of pure standard. 3: for *m*/*z* 61.028—dominant peak is acetic acid based on fastGC-PTR-ToF-MS, but there is a small peak for ethyl acetate fragment. 4: for *m*/*z* 89.060—dominant peak is ethyl acetate based on fastGC-PTR-ToF-MS, but there is a small peak for ethyl butanoate fragment.

**Table 4:** Main and fragment ions checked for *m*/*z* 89.060 in fastGC-PTR-ToF-MS.

Compound Name	Molecular Formula	Main/fragment Ions Checked			
		*m*/*z*	*m*/*z*	*m*/*z*	*m*/*z*
Ethyl acetate	C_4_H_8_O_2_	89.060 (C_4_H_8_O_2_)H^+^	61.028 (C_2_H_4_O_2_)H^+^	43.018 (C_2_H_3_O)H^+^	
Butyric acid	C_4_H_8_O_2_	89.060 (C_4_H_8_O_2_)H^+^	71.049 (C_4_H_6_O)H^+^	43.054 (C_3_H_7_)H^+^	29.039 (C_2_H_5_)H^+^

**Table 5:** Flavour standards checked in fastGC PTR-ToF-MS.

No	Flavour Standards	Molecular Formula	Molecular Weight	RI	RT (s)	Main/Fragment Ions Checked
1	Ethyl acetate	C_4_H_8_O_2_	88	888	58	89.060, 61.028, 43.018
2	2-Butanone	C_4_H_8_O	72	918	60	73.065
3	Ethanol	C_2_H_6_O	46	932	59	47.049
4	Ethyl butanoate	C_6_H_12_O_2_	116	1023	68	117.091, 89.060, 43.054
5	2-Methyl propanol	C_4_H_10_O	74	1092	69	57.07
6	2-Hexanone	C_6_H_12_O	100	1100	78	101.096
7	2-Heptanone	C_7_H_14_O	114	1182	84.5	115.112
8	3-Methyl butanol	C_5_H_12_O	88	1209	82.2	71.086
9	Ethyl hexanoate	C_8_H_16_O_2_	144	1233	89	145.122, 117.091
10	2-Nonanone	C_9_H_18_O	142	1390	109	143.143
11	Ethyl octanoate	C_10_H_20_O_2_	172	1435	111.5	127.112, 145.122
12	Acetic acid	C_2_H_4_O_2_	60	1449	112.5	61.028, 43.018
13	Benzaldehyde	C_7_H_6_O	106	1520	115.5	107.049
14	Ethyl decanoate	C_12_H_24_O_2_	200	1638	146	201.233, 155.107
15	Phenylethyl alcohol	C_8_H_10_O	122	1906	218	105.070

## Data Availability

Data are contained within the article and Supplementary Materials.

## Supplementary Materials

The following supporting information can be downloaded at: https://www.mdpi.com/article/10.3390/molecules29143275/s1, Table S1: The VOCS (*m*/*z*) detected by PTR-ToF-MS after LB672 fermentation that significantly (*p* < 0.05) distinguished between defined medium compositions (carbon sources) (M), fermentation time (0 and 7 days) (T) and temperature (either at 25 or 35 °C) (Temp), and their interaction effects., Table S2: The mean concentration (ppbV) of selected VOCs (*m*/*z*) after 7 days of fermentation by LB672 across defined medium compositions (DM: glucose added, DMFr: fructose added) at either 25 or 35 °C. Values are presented as mean ± standard error (*n* = 4).
