# Supplementary material for: Impact of Different Carbon Sources on Volatile Organic Compounds (VOCs) Produced during Fermentation by Levilactobacillus brevis WLP672 Measured Using Proton Transfer Reaction Time-of-Flight Mass Spectrometry (PTR-ToF-MS)
Source: Molecules. 2024 Jul 11;29(14):3275. doi: 10.3390/molecules29143275 (PMC11279293; doi:10.3390/molecules29143275)
Supplement: Supplementary file 1 [file molecules-29-03275-s001.zip › molecules-3045648-supplementary.pdf]

Table S1. The VOCS (*m/z*) detected by PTR-ToF-MS after LB672 fermentation that significantly ($p < 0.05$) distinguished between defined medium compositions (carbon sources) (M), fermentation time (0 and 7 days) (T) and temperature (either at 25 or 35 °C) (Temp), and their interaction effects.

No	*m/z*	Sum formula (Protonated ion)	Tentative identification	P value						
				M	T	Temp	M*T	M*Temp	T*Temp	M*T*Temp
1	26.016	$C_2H_2^+$	Common fragment	<0.0001	<0.0001	<0.0001	<0.0001	<0.0001	<0.0001	<0.0001
2	27.025	$C_2H_3^+$		<0.0001	<0.0001	<0.0001	<0.0001	<0.0001	<0.0001	<0.0001
3	28.031	$C_2H_4^+$		<0.0001	<0.0001	<0.0001	<0.0001	<0.0001	<0.0001	<0.0001
4	31.018	CH_2OH^+	Formaldehyde fragment	<0.0001	<0.0001	<0.0001	<0.0001	<0.0001	<0.0001	<0.0001
5	33.033	CH_4OH^+		0.969	<0.0001	<0.0001	0.005	0.001	<0.0001	0.003
6	34.996	H_2SH^+	Hydrogen sulfide	<0.0001	<0.0001	<0.0001	<0.0001	0.008	<0.0001	0.012
7	41.039	$C_3H_5^+$		<0.0001	<0.0001	<0.0001	<0.0001	0.005	<0.0001	<0.0001
8	42.010			<0.0001	<0.0001	<0.0001	<0.0001	<0.0001	<0.0001	<0.0001
9	43.018	$C_2H_3O^+$	Common fragment	<0.0001	<0.0001	<0.0001	<0.0001	<0.0001	<0.0001	<0.0001
10	43.054	$C_3H_7^+$	Propanol fragment*	<0.0001	<0.0001	<0.0001	<0.0001	<0.0001	<0.0001	<0.0001
11	45.033	$C_2H_4OH^+$	Acetaldehyde	<0.0001	<0.0001	<0.0001	<0.0001	<0.0001	<0.0001	<0.0001
12	47.049	$C_2H_6OH^+$	Ethanol*	<0.0001	<0.0001	<0.0001	<0.0001	<0.0001	<0.0001	<0.0001
13	49.011	CH_4SH^+	Methanethiol	<0.0001	<0.0001	<0.0001	<0.0001	0.037	<0.0001	0.057
14	53.006			<0.0001	<0.0001	<0.0001	<0.0001	<0.0001	<0.0001	<0.0001
15	53.040	$C_4H_5^+$		0.001	<0.0001	<0.0001	<0.0001	<0.0001	<0.0001	<0.0001
16	55.057	$C_4H_7^+$		0.986	<0.0001	0.276	0.009	0.723	0.422	0.668
17	57.036	$C_3H_4OH^+$		0.885	<0.0001	<0.0001	<0.0001	<0.0001	<0.0001	<0.0001
18	57.070	$C_4H_9^+$	1-Butanol fragment*	<0.0001	<0.0001	<0.0001	0.242	<0.0001	<0.0001	0.066
19	59.049	$C_3H_6OH^+$	Acetone*	<0.0001	<0.0001	<0.0001	<0.0001	<0.0001	0.710	0.092
20	61.028	$C_2H_4O_2H^+$	Acetic acid*	0.324	<0.0001	<0.0001	0.036	<0.0001	<0.0001	0.001
21	63.009	$CO_2{*}H_3O^+$		<0.0001	<0.0001	0.190	<0.0001	0.122	0.200	0.128

22	63.944			0.082	<0.0001	<0.0001	0.144	0.200	<0.0001	0.426
23	64.005			<0.0001	<0.0001	<0.0001	<0.0001	0.032	<0.0001	0.062
24	68.995			0.009	0.054	0.548	0.025	0.088	0.005	0.068
25	69.035	$C_4H_4OH^+$	Furan	0.744	<0.0001	<0.0001	0.071	0.459	0.038	0.111
26	69.070	$C_5H_9^+$	3-Methyl-3-buten-1-ol*	<0.0001	<0.0001	0.074	<0.0001	0.038	0.003	0.040
27	71.049			<0.0001	<0.0001	<0.0001	<0.0001	0.003	0.050	0.046
28	71.085	$C_5H_{11}^+$	3-Methyl-butanol fragment*	<0.0001	<0.0001	<0.0001	0.998	0.183	0.004	0.540
29	73.029	$C_3H_4O_2H^+$	2-Propenoic acid	0.613	<0.0001	0.006	0.114	0.572	0.096	0.994
30	73.064	$C_4H_8OH^+$	Butanal*	<0.0001	<0.0001	0.514	<0.0001	0.865	0.001	0.007
31	75.043	$C_3H_6O_2H^+$	Propanoic acid	0.070	<0.0001	<0.0001	<0.0001	0.018	<0.0001	0.032
32	77.011			0.002	<0.0001	0.001	0.045	0.425	<0.0001	0.905
33	77.030			0.004	<0.0001	0.196	0.051	0.009	0.465	0.005
34	78.967	$CH_2S_2H^+$		0.006	<0.0001	<0.0001	0.019	0.090	<0.0001	0.148
35	81.016			<0.0001	<0.0001	<0.0001	<0.0001	<0.0001	<0.0001	<0.0001
36	81.041	$C_4H_4N_2H^+$	Pyrazine*	0.046	<0.0001	<0.0001	0.036	0.566	0.732	0.868
37	81.070	$C_6H_9^+$		0.405	<0.0001	0.043	0.165	0.826	0.002	0.507
38	83.069			<0.0001	<0.0001	<0.0001	<0.0001	<0.0001	<0.0001	<0.0001
39	85.066	$C_5H_8OH^+$	2-Methyl-2-butenal* and 3-Methyl-2-butenal*	<0.0001	<0.0001	<0.0001	<0.0001	<0.0001	<0.0001	<0.0001
40	87.043	$C_4H_6O_2H^+$	Diacetyl	<0.0001	<0.0001	<0.0001	<0.0001	<0.0001	<0.0001	<0.0001
41	87.080	$C_5H_{10}OH^+$	2-Methyl butanal* and 3-Methyl butanal*	<0.0001	<0.0001	0.219	<0.0001	0.089	0.008	0.147
42	89.060	$C_4H_8O_2H^+$	Ethyl acetate*	<0.0001	<0.0001	<0.0001	<0.0001	<0.0001	<0.0001	<0.0001
43	91.027	$C_3H_6OSH^+$	Methyl thiolacetate/ Mercaptoacetone	<0.0001	<0.0001	<0.0001	<0.0001	<0.0001	<0.0001	<0.0001
44	91.072	$C_4H_{10}O_2H^+$	2,3-Butanediol*	<0.0001	<0.0001	<0.0001	<0.0001	<0.0001	<0.0001	<0.0001

45	93.996			<0.0001	<0.0001	<0.0001	0.079	0.365	<0.0001	0.365
46	94.037	$C_5[13]CH_4OH^+$		0.016	<0.0001	0.049	0.008	0.364	0.003	0.438
47	95.004	$C_2H_6S_2H^+$	Dimethyl disulfide*	0.004	<0.0001	<0.0001	0.010	0.031	<0.0001	0.050
48	95.093			<0.0001	<0.0001	<0.0001	<0.0001	<0.0001	<0.0001	<0.0001
49	97.033	$C_5H_4O_2H^+$	Furfural*	0.004	0.264	0.007	0.026	0.383	0.061	0.156
50	97.063	$C_6H_8OH^+$	2,5-Dimethylfuran/ Cyclohexen-2-one	<0.0001	<0.0001	<0.0001	0.144	<0.0001	<0.0001	<0.0001
51	97.106	$C_7H_{13}^+$		0.003	0.052	0.043	0.002	0.735	0.013	0.735
52	99.079	$C_6H_{10}OH^+$		<0.0001	<0.0001	<0.0001	<0.0001	<0.0001	<0.0001	<0.0001
53	99.119	$C_7H_{15}^+$	2-Heptanol fragment*	0.059	<0.0001	<0.0001	0.076	0.700	<0.0001	0.447
54	101.060	$C_5H_8O_2H^+$	2-Propenyl acetate	<0.0001	<0.0001	<0.0001	<0.0001	0.264	<0.0001	0.012
55	101.096	$C_6H_{12}OH^+$	2-Methyl pentanal*	0.078	<0.0001	0.511	0.431	0.567	0.642	0.295
56	103.074	$C_5H_{10}O_2H^+$	C5 esters and acids (pentanoic acid/3-methyl-butanoic acid)	0.027	<0.0001	<0.0001	<0.0001	0.017	0.544	0.002
57	105.046	$C_4H_8OSH^+$	Methional*	0.345	0.599	0.363	<0.0001	0.036	0.953	0.076
58	105.081			<0.0001	<0.0001	<0.0001	<0.0001	0.039	0.007	0.897
59	107.044	$C_7H_6OH^+$	Benzaldehyde*	<0.0001	<0.0001	<0.0001	<0.0001	0.013	0.093	<0.0001
60	107.066	$C_4H_{10}OSH^+$	Methionol*	<0.0001	<0.0001	<0.0001	<0.0001	<0.0001	<0.0001	<0.0001
61	107.107			<0.0001	<0.0001	<0.0001	<0.0001	<0.0001	<0.0001	<0.0001
62	109.059	$C_7H_8OH^+$	Benzyl alcohol*	<0.0001	<0.0001	<0.0001	<0.0001	<0.0001	<0.0001	<0.0001
63	109.088			0.823	0.269	0.014	0.001	0.057	0.204	0.019
64	111.044	$C_6H_6O_2H^+$		0.502	<0.0001	0.003	0.087	0.613	0.056	0.192
65	111.099			<0.0001	<0.0001	<0.0001	<0.0001	<0.0001	<0.0001	<0.0001
66	113.028	$C_5H_4O_3H^+$		0.204	<0.0001	0.022	0.633	0.677	0.053	0.484
67	113.061	$C_6H_8O_2H^+$		0.004	0.391	0.600	0.031	0.129	0.683	0.584
68	113.108	$C_6H_{12}N_2H^+$		0.880	<0.0001	0.031	0.301	0.221	0.391	0.159
69	115.040	$C_5H_6O_3H^+$		0.325	<0.0001	0.804	0.451	0.325	0.517	0.670
70	115.075	$C_6H_{10}O_2H^+$		0.447	0.341	0.036	0.051	0.307	0.515	0.665

71	115.112	$C_7H_{14}OH^+$	2-Heptanone*	0.916	0.009	0.004	0.069	0.717	0.023	0.256
72	117.048	$C_6H_9ClH^+$		0.908	<0.0001	0.249	0.243	0.566	0.646	0.283
73	117.091	$C_6H_{12}O_2H^+$	Hexanoic acid*	<0.0001	<0.0001	<0.0001	<0.0001	<0.0001	<0.0001	<0.0001
74	119.093	$C_6H_{14}SH^+$		<0.0001	0.123	0.110	<0.0001	0.096	<0.0001	0.044
75	121.057	$C_8H_8OH^+$	Benzeneacetaldehyde*	<0.0001	<0.0001	<0.0001	<0.0001	<0.0001	<0.0001	<0.0001
76	121.119			<0.0001	0.070	<0.0001	<0.0001	<0.0001	<0.0001	<0.0001
77	123.045	$C_7H_6O_2H^+$	Benzoic acid	0.006	<0.0001	0.201	0.002	0.004	0.257	0.005
78	123.117	$C_9H_{15}^+$		0.278	0.778	0.034	0.434	0.581	0.211	0.402
79	126.967			<0.0001	<0.0001	<0.0001	<0.0001	0.004	<0.0001	0.004
80	127.050			0.240	0.003	0.660	0.511	0.402	0.621	0.079
81	127.111	$C_8H_{14}OH^+$		0.585	<0.0001	<0.0001	<0.0001	0.261	0.020	0.013
82	129.047	$C_7H_9ClH^+$		0.960	0.896	0.222	0.019	0.636	0.785	0.580
83	129.090	$C_7H_{12}O_2H^+$		0.837	0.628	0.902	0.253	0.915	0.915	0.539
84	131.065	$C_7H_{11}ClH^+$ / $C_6H_{10}O_3H^+$		0.519	0.886	0.455	0.110	0.537	0.997	0.251
85	131.105	$C_7H_{14}O_2H^+$	Isoamyl acetate*	<0.0001	<0.0001	<0.0001	<0.0001	<0.0001	<0.0001	<0.0001
86	133.049			0.467	0.111	0.286	0.033	0.323	0.199	0.375
87	133.117	$C_7H_{16}O_2H^+$		0.645	0.895	0.630	0.458	0.861	0.742	0.526
88	135.100	$C_6H_{14}O_3H^+$		<0.0001	<0.0001	<0.0001	<0.0001	<0.0001	<0.0001	<0.0001
89	135.134			0.315	0.033	<0.0001	0.044	0.285	0.012	0.250
90	139.064			<0.0001	0.009	0.005	0.002	<0.0001	0.005	0.001
91	139.137			<0.0001	0.598	<0.0001	0.119	<0.0001	<0.0001	<0.0001
92	141.119			0.410	0.070	0.794	0.955	0.266	0.658	0.206
93	143.137	$C_9H_{18}OH^+$		0.237	0.969	0.621	0.044	0.393	0.037	0.262
94	145.123	$C_8H_{16}O_2H^+$	Octanoic acid*	0.001	<0.0001	0.001	0.003	0.018	0.022	0.006
95	147.070			0.217	0.007	0.554	0.074	0.809	0.860	0.382
96	149.046	$C_9H_8SH^+$		0.815	0.779	0.504	0.411	0.494	0.938	0.369
97	153.057	$C_8H_8O_3H^+$		0.229	0.124	0.156	0.780	0.314	0.391	0.025
98	159.138	$C_9H_{18}O_2H^+$	Nonanoic acid	0.004	<0.0001	0.694	0.199	0.844	0.375	0.900
99	163.077	$C_{10}H_{10}O_2H^+$		0.263	0.976	0.867	0.805	0.373	0.462	0.882

100	171.164			0.248	0.893	0.360	0.732	0.136	0.515	0.876
101	173.154	$C_{10}H_{20}O_2H^+$	Decanoic acid*	<0.0001	<0.0001	0.001	<0.0001	0.003	<0.0001	<0.0001
102	201.185	$C_{12}H_{24}O_2H^+$	Decanoic acid ethyl ester	<0.0001	<0.0001	<0.0001	<0.0001	<0.0001	0.001	<0.0001
103	207.043			0.474	0.001	0.673	0.850	0.110	0.539	0.337
104	223.063	$C_9H_{18}S_3H^+$		0.234	<0.0001	0.814	0.845	0.068	0.606	0.388

*: The *m/z* that HS-SPME-GC-MS identified.

Table S2. The mean concentration (ppbV) of selected VOCs (*m/z*) after 7 days of fermentation by LB672 across defined medium compositions (DM: glucose added, DMFr: fructose added) at either 25 or 35 °C. Values are presented as mean ± standard error (n = 4).

**No**	***m/z***	**DMFr**		**DM**	
		at 25 °C	at 35 °C	at 25 °C	at 35 °C
1	26.016	40.848 ± 1.043	49.628 ± 0.804	167.048 ± 1.866	441.51 ± 7.456
2	27.025	20.27 ± 0.688	23.736 ± 0.255	80.096 ± 1.173	191.378 ± 4.387
3	28.031	2.805 ± 0.104	3.686 ± 0.053	8.817 ± 0.16	21.155 ± 0.506
4	31.018	247.704 ± 6.033	291.079 ± 3.32	928.421 ± 8.446	2132.831 ± 30.714
5	33.033	161.498 ± 5.96	189.718 ± 5.257	140.657 ± 1.556	241.392 ± 10.145
6	34.996	2.77 ± 0.231	5.634 ± 0.647	7.906 ± 0.248	14.439 ± 1.055
7	41.039	34.928 ± 0.381	78.091 ± 0.524	21.498 ± 0.417	53.09 ± 1.581
8	42.01	52.428 ± 1.297	63.96 ± 0.768	205.561 ± 2.277	547.972 ± 10.345
9	43.018	3649.676 ± 142.383	3918.01 ± 147.166	4158.139 ± 62.515	9122.072 ± 343.959
10	43.054	29.115 ± 0.368	68.939 ± 3.343	5.054 ± 1.142	18.602 ± 1.923
11	45.033	612.53 ± 13.943	744.128 ± 8.842	2117.752 ± 20.246	4698.161 ± 145.303
12	47.049	4271.207 ± 108.356	4994.534 ± 72.983	17447.171 ± 176.659	44863.183 ± 1176.331
13	49.011	96.485 ± 13.064	289.972 ± 13.558	182.917 ± 13.601	459.487 ± 31.33
14	53.006	5.964 ± 0.09	11.472 ± 0.132	9.648 ± 0.13	26.73 ± 0.477

15	55.057	22.474 ± 1.083	21.268 ± 1.16	24.716 ± 1.962	25.263 ± 1.334
16	57.036	5.797 ± 0.038	8.598 ± 0.073	5.489 ± 0.101	11.73 ± 0.385
17	57.07	21.753 ± 0.217	50.568 ± 1.39	13.976 ± 0.558	33.623 ± 1.158
18	61.028	5341.215 ± 285.413	5452.21 ± 215.102	4389.825 ± 78.721	7640.963 ± 491.024
19	63.009	36.836 ± 0.751	32.916 ± 1.676	82.849 ± 1.968	127.534 ± 30.435
20	63.944	0.328 ± 0.035	1.389 ± 0.169	0.408 ± 0.057	1.748 ± 0.174
21	64.005	0.987 ± 0.037	1.502 ± 0.041	1.989 ± 0.124	3.025 ± 0.197
22	71.085	11.741 ± 0.282	23.249 ± 0.555	10.255 ± 0.532	21.316 ± 0.632
23	77.011	1.076 ± 0.088	1.548 ± 0.135	0.816 ± 0.047	1.214 ± 0.108
24	77.03	0.646 ± 0.072	0.523 ± 0.023	0.625 ± 0.042	0.88 ± 0.036
25	78.967	3.637 ± 0.436	13.713 ± 1.623	5.29 ± 0.533	20.076 ± 2.174
26	81.016	0.743 ± 0.024	0.693 ± 0.061	3.575 ± 0.167	11.114 ± 0.177
27	81.041	2.108 ± 0.148	2.419 ± 0.065	1.693 ± 0.068	2.127 ± 0.203
28	83.069	2.091 ± 0.083	2.852 ± 0.06	7.43 ± 0.127	22.269 ± 0.643
29	89.06	39.208 ± 2.117	41.4 ± 0.825	107.588 ± 1.683	583.1 ± 11.549
30	91.027	1.926 ± 0.068	5.259 ± 0.442	3.1 ± 0.159	14.864 ± 0.691
31	91.072	0.788 ± 0.039	0.839 ± 0.066	4.743 ± 0.19	27.938 ± 1.739
32	93.996	0.94 ± 0.06	2.12 ± 0.148	1.386 ± 0.053	2.352 ± 0.138
33	95.004	8.058 ± 0.768	24.239 ± 3.001	10.25 ± 0.808	37.968 ± 4.044
34	95.093	0.265 ± 0.039	0.534 ± 0.076	1.81 ± 0.071	12.486 ± 0.428
35	97.063	1.024 ± 0.116	4.976 ± 0.704	0.963 ± 0.088	10.732 ± 0.679
36	99.119	0.101 ± 0.025	0.29 ± 0.014	0.059 ± 0.025	0.239 ± 0.014
37	103.074	0.541 ± 0.025	0.597 ± 0.013	0.633 ± 0.066	0.997 ± 0.054
38	107.066	8.154 ± 0.728	10.286 ± 0.575	27.644 ± 1.292	143.431 ± 10.149
39	107.107	0.676 ± 0.04	0.763 ± 0.087	2.129 ± 0.124	7.99 ± 0.51
40	109.059	0.024 ± 0.013	0.095 ± 0.01	0.336 ± 0.016	2.186 ± 0.109
41	111.099	0.192 ± 0.034	0.231 ± 0.011	0.46 ± 0.02	2.347 ± 0.052
42	115.112	0.09 ± 0.016	0.212 ± 0.011	0.121 ± 0.025	0.283 ± 0.088

43	117.091	0.375 ± 0.007	0.418 ± 0.007	0.488 ± 0.032	1.118 ± 0.054
44	121.057	1.655 ± 0.17	1.74 ± 0.105	1.841 ± 0.056	5.062 ± 0.345
45	121.119	0.024 ± 0.022	0 ± 0	0.076 ± 0.014	0.448 ± 0.032
46	123.045	1.036 ± 0.072	0.749 ± 0.041	1.05 ± 0.106	1.323 ± 0.048
47	126.967	0.415 ± 0.011	0.823 ± 0.056	0.708 ± 0.045	1.55 ± 0.112
48	131.105	0.372 ± 0.025	0.383 ± 0.026	0.344 ± 0.02	0.67 ± 0.031
49	135.1	0.119 ± 0.02	0.123 ± 0.009	0.265 ± 0.018	2.334 ± 0.028
50	135.134	0.007 ± 0.007	0.062 ± 0.018	0.017 ± 0.007	0.126 ± 0.04
51	139.064	0.086 ± 0.01	0.064 ± 0.018	0.108 ± 0.011	0.25 ± 0.017
52	145.123	0.192 ± 0.024	0.208 ± 0.023	0.218 ± 0.033	0.43 ± 0.047
53	173.154	0.089 ± 0.016	0.106 ± 0.008	0.189 ± 0.008	0.629 ± 0.108
54	201.185	0.032 ± 0.009	0.022 ± 0.009	0.046 ± 0.013	0.177 ± 0.019
